# A predictive model for vertebrate bone identification from collagen using proteomic mass spectrometry

**DOI:** 10.1038/s41598-021-90231-5

**Published:** 2021-05-25

**Authors:** Heyi Yang, Erin R. Butler, Samantha A. Monier, Jennifer Teubl, David Fenyö, Beatrix Ueberheide, Donald Siegel

**Affiliations:** 1 Office of Chief Medical Examiner, 421 East 26th Street, New York, NY 10016 USA; 2 Institute for Systems Genetics, Department of Biochemistry and Molecular Pharmacology, NYU Grossman School of Medicine, New York, NY 10016 USA; 3 Department of Biochemistry and Molecular Pharmacology, Department of Neurology, Director Proteomics Laboratory, Division of Advanced Research Technologies, NYU Grossman School of Medicine, New York, NY 10016 USA

**Keywords:** Mass spectrometry, Proteomic analysis, Machine learning, Proteome informatics, Proteomics

## Abstract

Proteogenomics is an increasingly common method for species identification as it allows for rapid and inexpensive interrogation of an unknown organism’s proteome—even when the proteome is partially degraded. The proteomic method typically uses tandem mass spectrometry to survey all peptides detectable in a sample that frequently contains hundreds or thousands of proteins. Species identification is based on detection of a small numbers of species-specific peptides. Genetic analysis of proteins by mass spectrometry, however, is a developing field, and the bone proteome, typically consisting of only two proteins, pushes the limits of this technology. Nearly 20% of highly confident spectra from modern human bone samples identify non-human species when searched against a vertebrate database—as would be necessary with a fragment of unknown bone. These non-human peptides are often the result of current limitations in mass spectrometry or algorithm interpretation errors. Consequently, it is difficult to know if a “species-specific” peptide used to identify a sample is actually present in that sample. Here we evaluate the causes of peptide sequence errors and propose an unbiased, probabilistic approach to determine the likelihood that a species is correctly identified from bone without relying on species-specific peptides.

## Introduction

**Figure 1:**
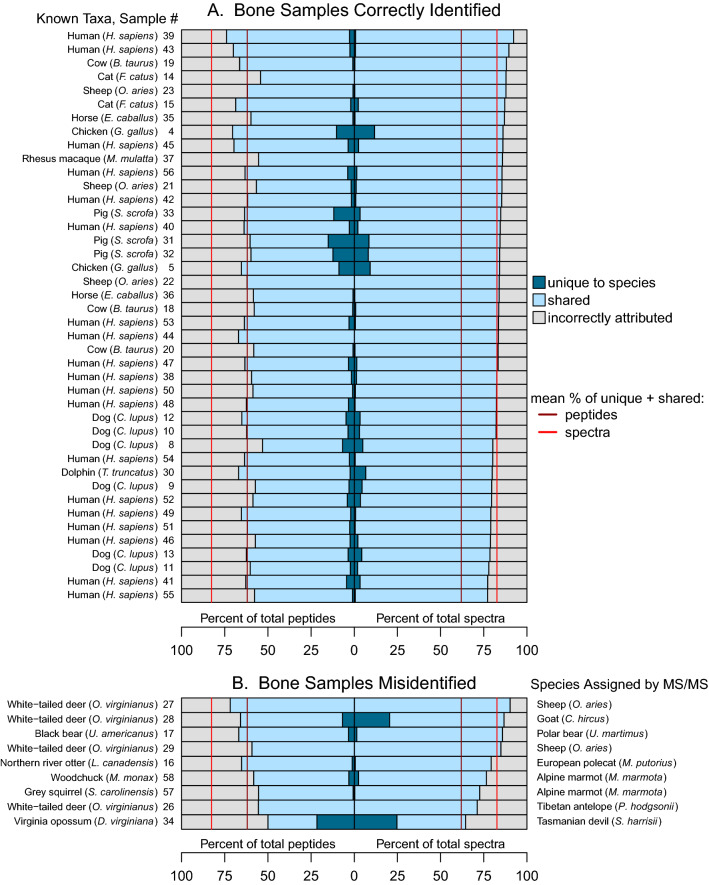
Proportion of peptides and spectra correctly and incorrectly attributed to sample species. Rows represent MS/MS data from each sample searched against an NR vertebrate database. (**A**) Sample species correctly identified. (**B**) Samples of known species misidentified due to poor database representation (Supplemental Table S5). Species assigned by MS/MS are listed on the right. Total number of peptides and spectra found for each sample can be found in Supplemental Table S9A.

Species identification of morphologically unidentifiable bones or bone fragments can prove challenging in paleontology, anthropology and forensics in the absence of DNA. Numerous methods have been proposed for species identification based on proteins using tandem mass spectrometry (MS)^1–3^. Like DNA, these methods rely on detecting genetic changes that occur during speciation. For proteins, these are amino acid differences detected by MS in confidently identified peptides. Most proteogenomic species identification methods seek to identify peptides that are unique to a single species or shared by a limited number of species^1–3^. While the two main criteria for proteomic species identification—confidently identified peptides and their relative uniqueness—can work when large numbers of different proteins are available for sample analysis^1^, they can be difficult standards to achieve with bone. Bone extraction commonly identifies only a few proteins with good peptide coverage^4^ (Supplemental Table S1). Typically, these are collagen 1 alpha 1 (COL1A1) and collagen 1 alpha 2 (COL1A2). These collagens are highly conserved over evolution^5^, have numerous repeating motifs and significant posttranslational modifications (typically proline hydroxylation)^6^ all of which tend to confound MS interpretation by offering multiple amino acid sequence possibilities^6–8^ (see below). The problem of determining species from an unknown bone is compounded as large protein databases must be searched (e.g. vertebrate or mammal) which inevitably leads to the identification of false positives resulting from the limitations of mass spectrometry (e.g. fragmentation efficiency) and interpreting algorithms^7,8^. Frequently 40% of confidently identified collagen peptides match species other than the sample species (Fig. 1). Indeed, it is not uncommon to find multiple, highly confident, different species-specific peptides in a single sample none of which match the sample species (Supplemental Tables S2A), S2B and S3). This large number of apparently random amino acid identifications (Supplemental Tables S4A, S4B) results in a random group of species identifications. Consequently, these inevitable, high confidence, false positive peptides, often representing a significant proportion of the limited number of collagen peptides identified (Supplemental Tables S3) makes using species specific peptides, or a limited number of shared species peptides, unfeasible as the false positives can be assigned to species other than the sample species. Data presented here demonstrate that an unbiased analysis of all highly confident collagen spectra (i.e. not weighting them for species specificity), using a logistic regression classifier, represents a new method for vertebrate bone taxonomic identification, as it does not employ comparing individual species databases^1^, but rather uses empirical data including the large numbers of false positives that are inevitably detected from single source samples. Analysis of 58 bone samples from 18 taxa demonstrate that using all accumulated spectra, regardless of assigned species, correctly identifies human samples 100% of the time and all species 82% of the time. A Bayesian logistic regression model predicts correct species assignments with 87.5% accuracy, and order assignments with 100% accuracy. As databases become more complete, accurate identification of more taxa to the species level with only limited peptide coverage will improve (Supplemental Table S5).

## Results

### Terms and definitions

- Bayesian models—three Bayesian models are used to evaluate the probabilities of correct species, order and human identifications from bone peptides.
- Datasets are the same MS data searched against non-redundant (NR) mammalian or vertebrate databases.
- Datatypes are peptides, peptide spectrum match (PSM) or spectra.
- Isobaric peptides are species consensus sequence peptides with one or more isobaric amino acid substitutions.
- Non-isobaric peptides are species consensus sequence peptides with one or more non-isobaric amino acid substitutions.

#### Frequency and source of MS interpretation errors

Tandem mass spectrometry (MS/MS) was performed on 58 bone samples of varied age and condition (Supplemental Table S6) representing 18 taxonomic groups [15 known species and seven unknown: 2 birds, 2 fish, 1 frog and 2 ovicaprid (goat or sheep)]. Data were searched against both vertebrate and mammalian databases. On average 260 spectra were identified per sample (median 239, range 79–710 from a vertebrate database search, Supplemental Table S7). The majority of spectra with E-values ≤ 0.01 (X! Tandem^9^) filtered for a false discovery rate (FDR) < 0.01, (~ 81%) identified a single highest scoring peptide (Supplemental Table S7). These peptides may or may not represent a single species, i.e. they may be shared between species (Supplemental Table S8). Nor do these peptides always identify the correct species, i.e. they may identify a species other than the sample species (Supplemental Table S3)^10^.

Approximately 17% of spectra identified two equally likely peptides (i.e. identical E-values), 1.6% three peptides and < 1% four peptides (Supplemental Table S7). (Biologically, there should be no more than two possible peptides for a heterozygous sample.) The left side of Fig. 1A demonstrates that while ~ 60% of identified peptides correctly match the sample species, the bulk of these peptides (~ 92%) are shared with other species (Supplemental Table S8) and importantly, ~ 40% of identified peptides do not match the sample species from which they came. Figure 1B shows that when a species has limited representation in a database (see also Supplemental Table S5) that nearly 60% of peptides can again be assigned to a species, but that species is not the sample species. Again, ~ 40% of identified peptides are assigned to species other than the top selected species. Interestingly, four samples of one species not well represented in the database, Virginia white-tailed deer (*Odocoileus virginianus*) (Fig. 1B), were incorrectly assigned to three different species—2 sheep, 1 goat and 1 Tibetan antelope (*Pantholops hodgsonii*). Consequently, the question arises: is there a method to confidently identify the species of an unknown bone sample when significant numbers of high-quality spectra identify peptides that match to multiple species other than the sample species?

#### Analysis of incorrectly assigned peptides

All peptides that identify species other than the actual species (~ 40%, Fig. 1A) typically differ from the peptide of the actual species by a single amino acid. These peptides may be divided into two groups, those that differ by an isobaric amino acid change, and those that differ by a non-isobaric amino acid change. The number and specific isobaric and non-isobaric substitutions for 14 human bone samples are listed in Supplemental Table S4B.

Isobaric peptides can result from isobaric amino acid substitutions (e.g., I ⇔ L), amino acid modifications (e.g., P(OH)A ⇔ PS)^6^, or adjacent or near adjacent amino acid inversions for which there are no fragment ions able to distinguish them (e.g., ~ PGS ~  ⇔ ~ SGP ~)^8^.

Non-isobaric peptides (i.e. peptides sequences that differ from the true species’ peptide by a non-isobaric amino acid substitution) may be true polymorphisms, or due to mass spectrometry limitations (e.g. variable/poor fragmentation, relatively low resolution and/or accuracy) or algorithm interpretation errors^7,8^. Biologically, some amino acid changes are more likely than others (see below). Consequently, by evaluating differences in spectra quality, it might be possible to distinguish true polymorphisms (presumably high-quality spectra) from artifacts (presumably low-quality spectra) and thereby improve species identification. All analyzed spectra had E-values ≤ 0.01 (X! Tandem^9^, see “Methods”).

Non-isobaric peptides may be divided into three categories: (i) those in which a single amino acid change results from a single nucleotide change, (ii) those in which a single amino acid change results from two or three nucleotide changes, and (iii) those peptides with more than one amino acid change. If non-isobaric peptides represent true polymorphisms, then, biologically, single nucleotide changes resulting in single amino acid changes would be expected to occur more commonly than two or more nucleotide changes^11^. Multiple amino acid changes in a single peptide would be even less common. Consequently, multiple nucleotide or amino acid changes may represent artifacts which in turn might be expected to have lower quality (E-values) scores.

**Figure 2:**
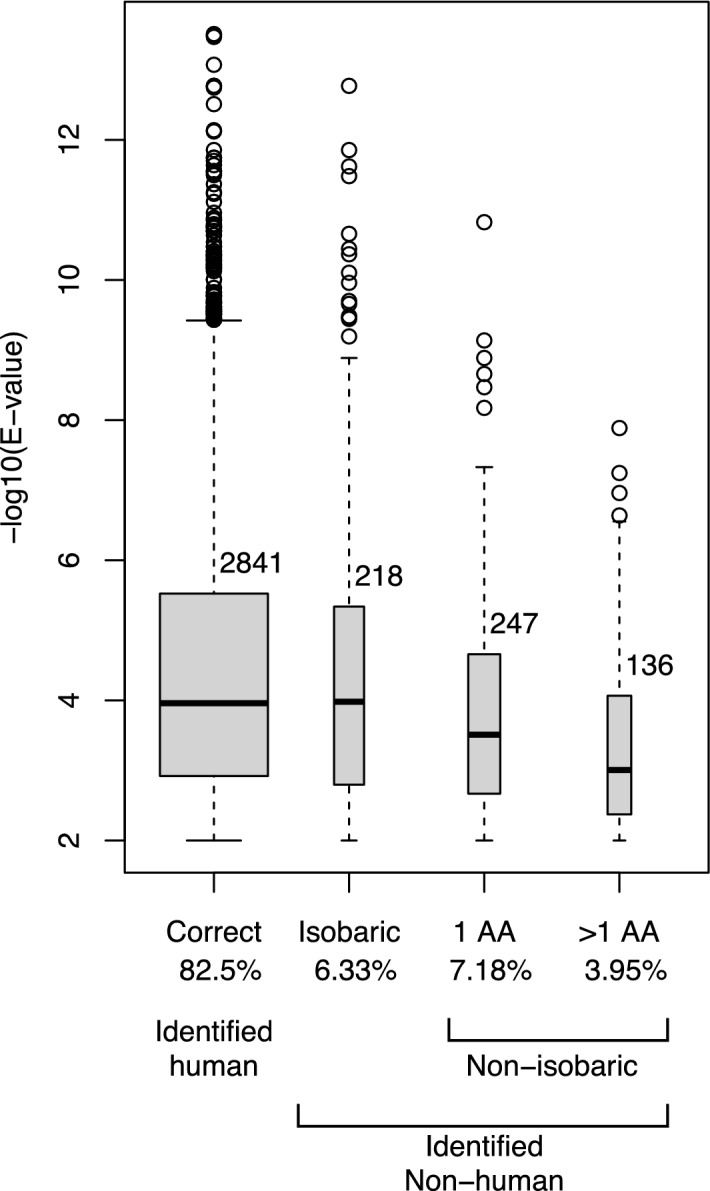
E-values of peptides identified by MS/MS in 19 human bone samples searched against a vertebrate database. Boxplots show distributions of E-values of spectra that matched to human peptides (correctly identified) (left) and spectra that matched only to non-human peptide sequences (incorrectly attributed). Width of boxes correspond to the number of spectra in each category, also printed above each box. Incorrectly attributed peptides differ from human consensus sequence peptides by one or more amino acids. These amino acid differences can be isobaric or non-isobaric. A post hoc Tukey test revealed correct peptides and isobaric peptides to be statistically similar and 1 AA and > 1 AA peptides also to be to be statistically similar. All other comparisons were significantly different (p < 0.01).

To determine if an E-value cutoff could be used to filter false positives, analyses of incorrectly identified peptides (i.e. peptides that identify a species other than the sample species) were evaluated. Figure 2 (see also Supplemental Table S4A) shows the number and E-values of spectra from 19 human bone samples searched against a vertebrate database. Approximately 83% of spectra matched to human identified peptides, and ~ 18% of spectra incorrectly identified only peptides from non-human species. Of the latter, ~ 36% are isobaric peptides, and 64% non-isobaric peptides. Median spectra E-values of isobaric peptides assigned to the wrong species were similar to E-values assigned to peptides of the correct species (Supplemental Table S4A), implying alternative algorithm interpretations for isobaric amino acid substitutions, modifications or rearrangements (see above). The majority of incorrectly identified non-isobaric peptides (64%) had single amino acid changes, while 36% had two or more amino acid changes. As suspected, non-isobaric peptides with single amino acid changes had lower median E-values than peptides assigned to the correct species. Those peptides with two or more amino acid changes (biologically less likely) had still lower median E-values (Supplemental Table S4A). The difference in E-values between spectra correctly assigned to a species, and non-isobaric spectra incorrectly assigned was statistically significant (p < 0.01, See Fig. 2). However, use of the median E-value of the latter as a possible filter to reduce false positives removed only 68 > 1 amino acid changed spectra, while eliminating 710 (~ 25%) of spectra correctly assigned. The loss of these spectra reduced the accuracy of species identification as they proved informative for distinguishing closely related species (see below). Consequently, use of an E-value filter did not prove beneficial.

#### Species identification using unique and shared peptides

The most obvious way of using the limited MS/MS data derived from bone to determine species would be to sum the unique peptides for each species identified in a sample and assume that the largest number of unique peptides most likely identifies the correct species. This method, however, turns out to be the least predictive—identifying correct taxa only about 40% of the time (Supplemental Tables S2A and S2B). One reason for this is that on occasion few or no peptides are unique to the sample species, or more peptides are unique to other species. For example, while 175 non-redundant peptides were identified in a cat bone (*Felis catus*), none were unique to cat (Supplemental Table S3/sample 14). However, 45 unique peptides were identified belonging to 29 different species.

Another method would be to sum the species of all peptides found in a sample (unique and shared) and assume that the most frequently identified species would likely be the correct species. This method uses significantly more data (on average ~ 5% of peptides are unique to a single species compared with ~ 60% unique plus shared) and increases correct species identification to ~ 83% (Supplemental Tables S2A and S2B).

#### Species identification using spectra

Although the peptide method described above is commonly used for species identification^1–3,6,12^, there is no a priori reason for identifying species based on peptides as opposed to using every spectra with a high-quality peptide assignment. Indeed, as shown in Fig. 1A (see also Supplemental Tables S9A and S9B) ~ 80% of spectra are assigned to the correct species, while only ~ 60% of peptides (Fig. 1A) are correctly assigned. There are several reasons for this. First, the spectra method increases the number of data points used for analysis as all spectra are counted, including redundant spectra. Redundant peptides are not counted by the peptide method (see below). Second, the spectral method diminishes the multiplier effect of generating and counting the multiple isobaric peptides that can be derived from a single spectrum (in the end only one peptide can be correct) effectively reducing the influence of false isobaric possibilities in determining species and increasing the percent of data correctly assigned (see example Supplemental Table S10).

To identify a sample’s species using the spectra method (Fig. 1A,B, right side), the ratios of all identified species spectra to total spectra are calculated (non-redundant (NR) vertebrate database). The “winner” species is the one with the highest ratio (Supplemental Table S11A). All samples from species well characterized in the database (42 representing 10 taxa) were correctly identified by the top species ratio (Fig. 1A right side and Supplemental Table S5). Two samples of uncertain species (ovicaprids) were identified as sheep. (All three known sheep samples were correctly identified. There were no known goat samples.) Two birds and two fish of unknown species were correctly identified to their lowest known taxonomic rankings (infraclass Neognathae for birds, class Actinopterygii for fish). An unknown frog species from the family Hylidae was identified to the correct order (Anura). The 9 misidentified samples (Fig. 1B), representing six species (four were Virginia white-tailed deer) had limited representation in the NR database (Supplemental Table S5).

**Figure 3:**
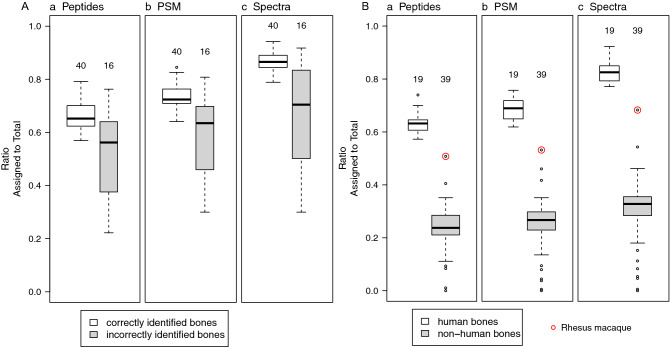
Comparison of peptide, PMS and spectra methods for determining species. (**A**) Comparison of distribution of ratios for correctly and incorrectly identified bone samples of known species, results from a mammal database search. (a) Non-redundant peptide/total peptides, (b) peptide-spectrum matches (PSM)—i.e. all (redundant) peptide identifications/total peptides, (c) all (redundant) spectra/total spectra. On average 77% of spectra were found two or more times with a mean of ~ 5.7 spectra for each sequence (median 3, range 6–55). As results are from a mammal database search, incorrectly identified samples include all seven non-mammal samples. Above each box plot is the total number of samples in each category. (**B**) Comparison of distribution of ratios (peptides or spectra assigned to human/total peptides or total spectra identified) for human and non-human bone samples using results from vertebrate database search. Descriptions of a–c same as (**A**). Rhesus macaque, the only non-human primate, is circled in red.

The advantage of using the spectra method may be seen in Fig. 3A which not only shows that a greater fraction of spectra are assigned to the correct species than the fraction of peptides (Fig. 3A, panel c vs a respectively), but that there is a greater spread between median values of correctly and incorrectly identified samples by the spectra method than by the peptide method. Importantly, even if redundant peptides are considered (Fig. 3A, panel b), making the peptide method more similar to the spectra method, the spectra method still shows the greatest fraction of correctly identified species and a greater spread between correctly and incorrectly identified species medians. Similar results are obtained when evaluating samples solely on the basis of the number of human spectra or peptides (Fig. 3B). Of note, the non-human sample with the greatest percentage of human spectra or peptides in this figure is macaque, which is easily distinguishable from human. Differences in the ability of peptide, peptide spectrum matches (PSM) and spectra methods to predict the accuracy of a species assignment of an unknown sample are described below.

**Figure 4:**
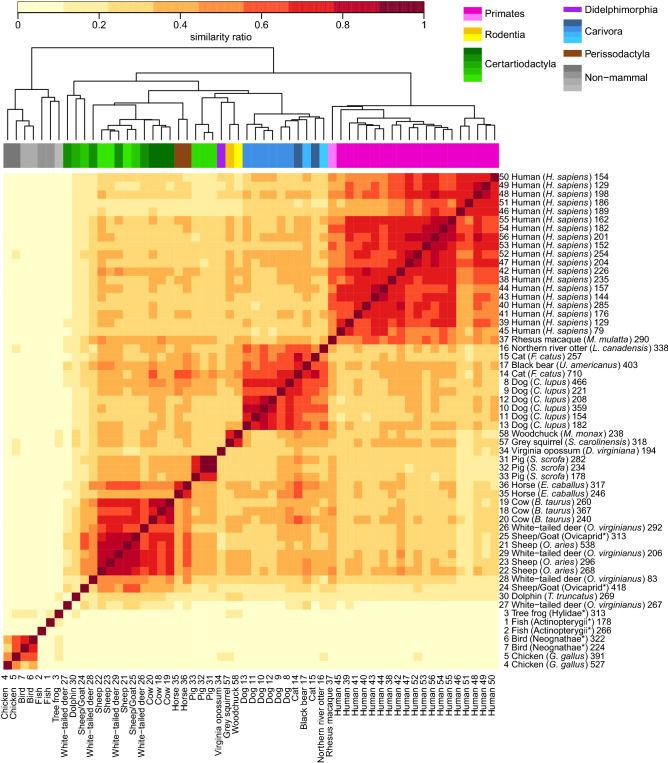
Heatmap and hierarchical clustering of pairwise comparison of bone sample spectra. Symmetric heat map of similarity ratios between all bones. Darker red indicates greater similarities between samples, light yellow indicates samples with a few or no spectra in common. Importantly, sample clustering coincides, for the most part, with evolutionarily similar taxa as seen in the dendrogram and taxa color-coded bar at top of figure. On the right axis the total number of spectra for each sample is listed along with common and Latin names of species, or most specific known taxa, indicated by * (see “Methods” for details).

To evaluate the ability to differentiate species based on spectra, unsupervised hierarchical clustering analysis was performed using MS/MS data searched against the NR vertebrate database. Clustering was based on a pairwise comparison of samples using all identified spectra (regardless of species assignment). Figure 4 is a symmetric heat map of similarity ratios between all bones, with darker shading demonstrating greater similarities. Importantly, sample clustering coincides, for the most part, with evolutionarily similar taxa which may be seen in the dendrogram and taxa color-coded bar at top of figure.

#### Influence of database size and the evolutionary conservation of collagens

Two important variables that influence accurate species identification are the number of proteins identified in a sample and the thoroughness of a species’ representation in a protein database^7,8^. In bone, only two proteins are routinely identified with good coverage, collagen types 1A1 and 1A2 (Supplemental Table S1)^4^. Evolutionary analysis of these proteins over 450 Myr shows that they share strong exon and intron homologies both between isoforms and across species^5^. Consequently, their ability to discriminate between closely related species is limited. Indeed, Fig. 1A shows that between ~ 75% and ~ 95% of spectra that correctly identify the sample from which they came are shared with other species (Supplemental Table S8). These problems, i.e. the limited number of proteins that are detected in bone and peptide homology between species, are compounded when a species has limited representation in the database as another species will inevitably be identified with multiple, high quality (E-value) spectra. For example, Virginia white-tailed deer with only 89 proteins entries in the NR vertebrate database (Supplemental Table S5) was most often identified as sheep which had 34,779 protein entries. Consequently, when trying to identify an unknown sample with a limited number of proteins, poor database representation, as well as the inevitable identification of spectra that do not match the true species (see above), a statistical method to determine the probability of correct species identifications is required.

#### Determining probabilities that samples are correctly assigned to species, order, or are of human origin

A Bayesian probabilistic logistic regression model was chosen for its relative stability with smaller and imbalanced datasets, as well as its ability to generate informative probability distributions rather than point estimates^13^. Bayesian models were fitted to processed search result data as described in “Methods” (see Supplemental Data for scripts). Depending on their purpose (determining species, order or human origin of a sample) the models were trained on different search results generated from the same raw MS data. The three Bayesian models were developed for:

1. species determination—a mammal database was searched
2. order determination—a mammal database was searched
3. human determination—a vertebrate database was searched

For analysis of the accuracy of both species and order matches, the mammalian database was chosen to deliberately generate incorrectly identified samples (i.e. the seven non-mammals, see Supplemental Table S9B and S9C) in order to train the model. Note, that for analysis of correct species matches, the two ovicaprids (sheet/goat) samples were removed from the dataset as their species of origin is unknown. The five non-mammal samples of unknown species (2 birds, 2 fish, and 1 frog) were still used since any species matches to a mammalian database search are incorrect. For determining whether a sample is human, search results from the vertebrate database were used. The “winner” species, order, or human origin result was defined as the one with the highest ratio of hits (either peptides, PSM, or spectra) for a single species or order or human result, to the total number of hits. The models were trained to determine if this ratio was predictive of whether the resulting top hit species or order or human result, matched the known species or order of origin of the sample. Each of the above three models were initially evaluated with respect to peptides, PSMs and spectra using Pareto-Smoothed Importance Sampling Leave-One-Out cross validation (PSIS-LOO) in order to estimate which of these datatypes give the highest prediction accuracy—spectra do (see below). Consequently, for final probability analysis, the exact leave one out cross validation method was performed using spectra.

##### Comparing peptide, PSM and spectra ratio datatypes using PSIS-LOO

**Figure 5:**
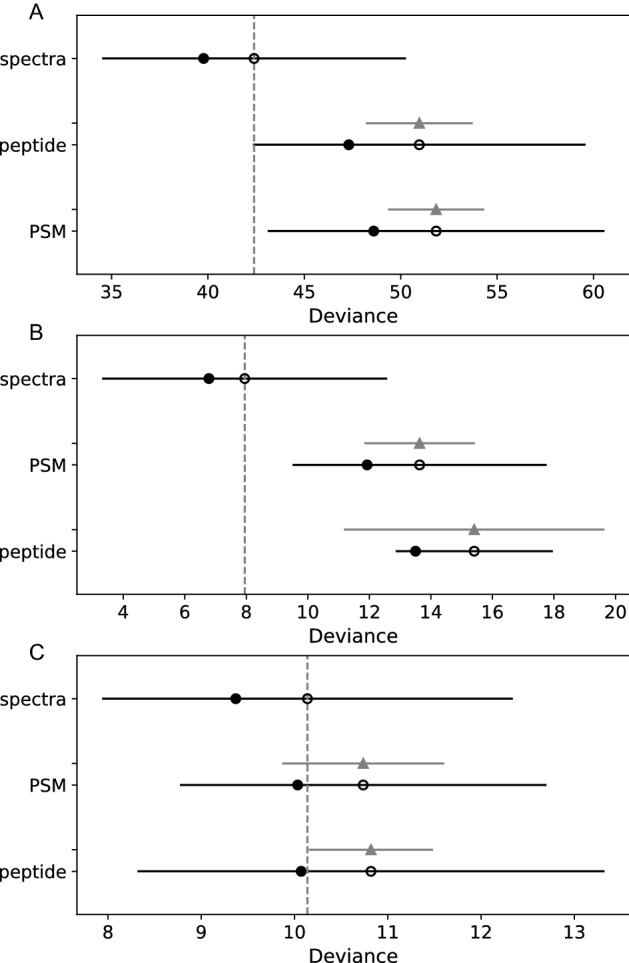
Comparison of peptide, PSM, and spectra models using PSIS-LOO. PSIS-LOO estimates comparing pointwise out-of-sample prediction accuracy demonstrate that the spectra method outperforms the PSM and peptide methods for species (**A**), order (**B**) and human identification (**C**). Solid circles are the in-sample deviance of each model, open circles are the PSIS-LOO estimate, and the solid dark line that passes through the open circle is the standard error of the estimate. The grey triangle and grey lines show the standard error of the difference between each PSIS-LOO and the top-ranked PSIS-LOO.

As mentioned, before evaluating the three Bayesian models (species, order and human origin) for their predictive abilities, it was first necessary to determine which datatype (peptides, PSM or spectra) gave the most accurate results. Therefore, each of the three models was trained separately with peptides, PSM, and spectra ratios and compared using a PSIS-LOO^14^ method of estimating out-of-sample prediction accuracy using within sample fits implemented using the python package PYMC3. Figure 5 (see also Supplemental Table S12) shows that the spectra method of organizing data had the best estimate of out-sample-accuracy (i.e. lowest deviance), particularly for species and order. As such, subsequent analyses were performed using spectra ratios.

##### Full leave-one-out cross validation with spectra ratios

In order to test the performance of each model (species, order, and human origin) on unseen data, exact leave-one-out cross validations were performed. Each model was iteratively trained on all spectra data except for one sample, then the trained model was used to generate the posterior predictive distribution of the unseen sample data. For all three types of search results (predicting species, order, and human origin), a receiver operating characteristic (ROC) curve was generated using the mean of the posterior predictive distribution for each unseen sample and the area under the curve (AUC) calculated.

**Figure 6:**
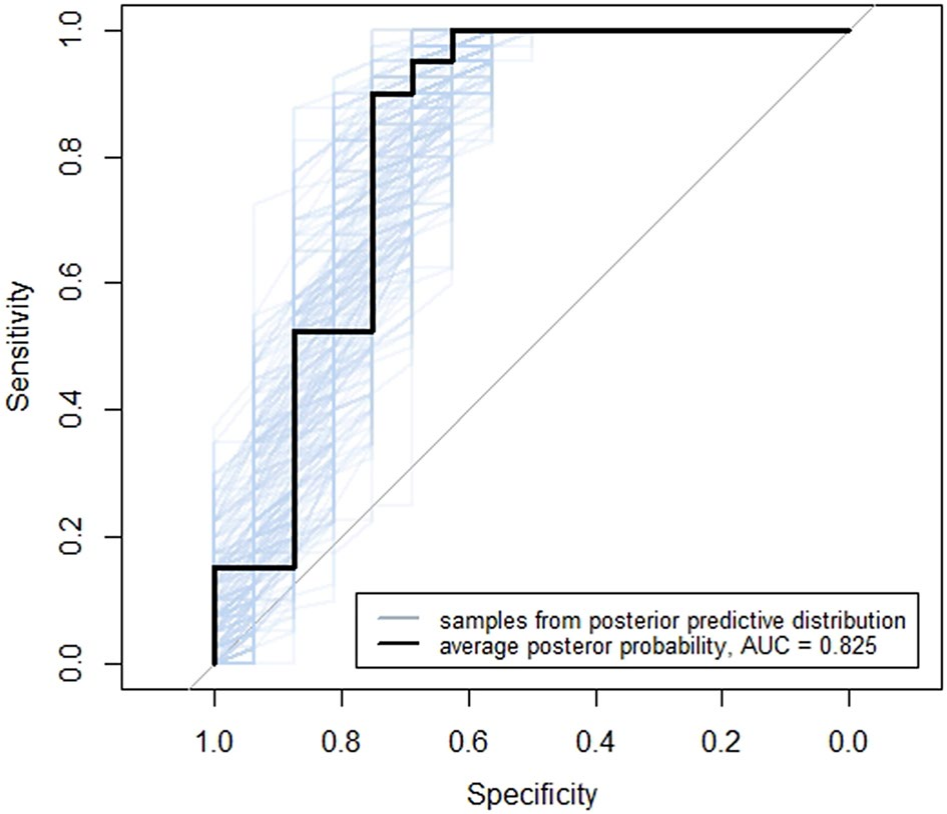
Receiver operating characteristic (ROC) curve of posterior probability of correct species assignment using spectra ratio from leave-one-out cross validation. Full leave-one-out cross validation with spectra data was performed and a ROC curve generated using the mean of the posterior predictive distribution for each unseen sample, and the area under the curve (AUC) calculated. For the prediction of whether the top hit species (by spectra ratio) was correct, the AUC was 0.825.

**Figure 7:**
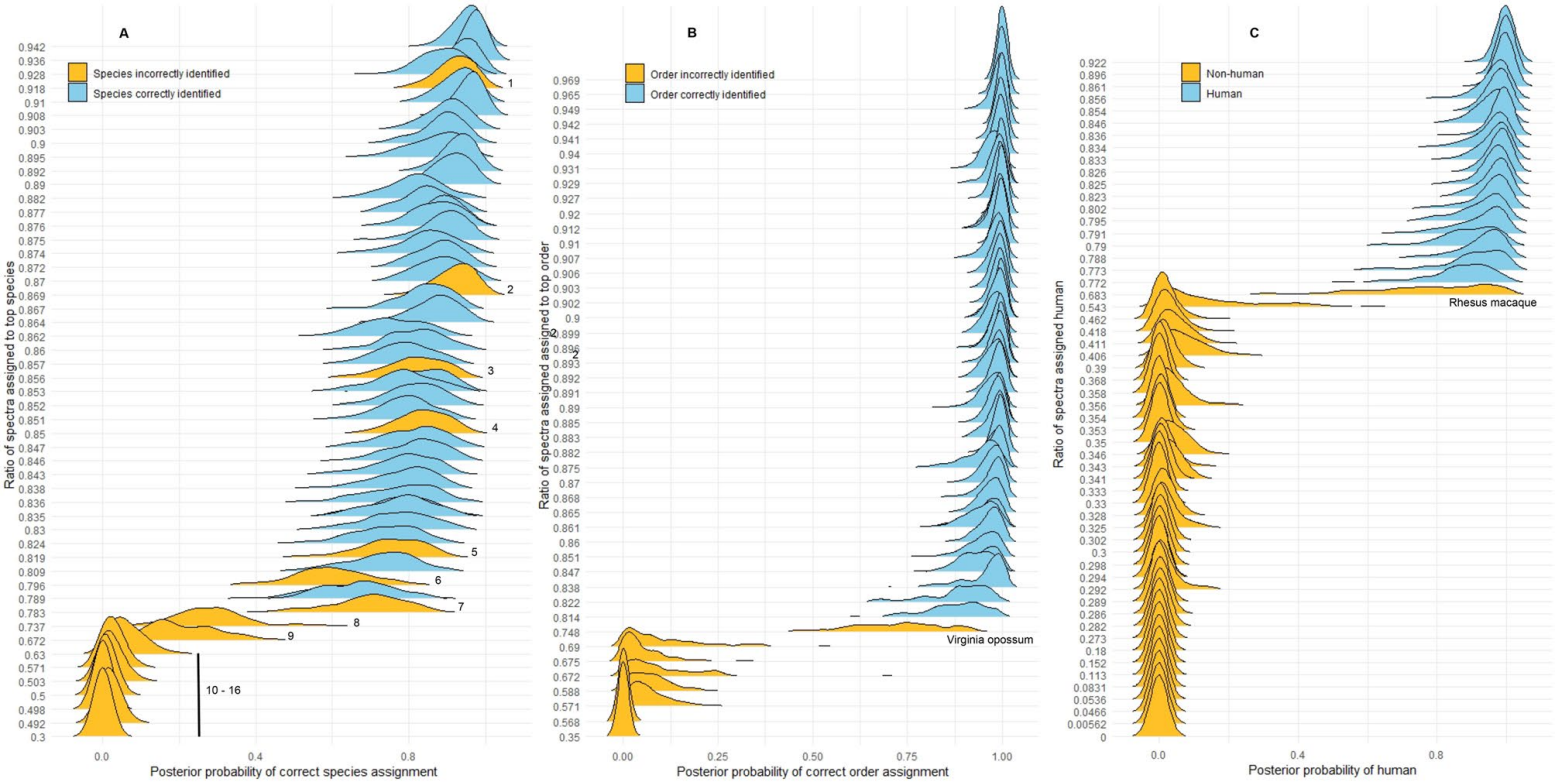
Predictive posterior distributions for species, order, and human origin of samples from exact leave-one-out cross validation. (**A**) Predictive posterior distributions of correct species assignment using spectra ratios from a mammalian database search. Wider, flatter distributions reflect greater uncertainty in the model for these values. Mammal bones for which species was incorrectly identified by spectra ratio are: 1, 2, 4, and 8 = Virginia white-tailed deer, 3 = brown bear, 5 = woodchuck, 6 = Northern river otter, 7 = grey squirrel, and 9 = Virginia opossum, all of which are species poorly represented in the database (See Supplemental Table S5). 10–16 are the seven non-mammals (see Supplemental Table S9). (**B**) Predictive posterior distributions of correct order assignment using spectra ratios from a mammalian database search. The seven non-mammalian samples were incorrectly identified by mammalian database search all have very low predicted probability of correct order assignment (bottom left, in orange). The orders of all mammals are correctly identified by spectra ratio except Virginia opossum (order Didelphimorphia), which was identified as order Dasyuromorphia. (**C**) Predictive posterior distributions of human origin using human spectra ratios from a vertebrate database search. Correctly identified human samples have narrow distributions with high predicted probability of human origin, and are clearly separated from non-human samples with very low probabilities. The only exception and the only non-human primate in the dataset, rhesus monkey, stretches from the bottom right (orange) to center of the figure.

• *Species prediction* For species prediction (using the spectra ratio method and mammalian database), the calculated receiver operating characteristic (ROC) AUC was 0.83 (Fig. 6). The mean probability of correct species identification in cross validation for known correct samples was 0.83 ± 0.10 and mean probability of correct species for the incorrectly identified samples 0.39 ± 0.38 (Fig. 7A). Of the 16 incorrectly identified samples seven were non-mammals and had a mean probability of correct identification of 0.02 ± 0.03. The remaining nine mammals [four deer and one each of black bear (*Ursus americanus*), grey squirrel (*Sciurus carolinensis*), northern river otter (*Lontra canadensis*), Virginia opossum (*Didelphis virginiana*) and woodchuck (*Marmota monax*)] were poorly represented in the mammalian database with typically less than a hundred protein entries compared to tens of thousands of entries for other mammals (Supplemental Table S5). The mean probability of correct identification of these mammalian samples was 0.70 ± 0.26 (Fig. 7A). The lowest mean probability of correct identification for a mammal was 0.21 for a Virginia opossum identified as a Tasmanian devil (*Sarcophilus harrisii*). Both animals are marsupials (Infraclass: Marsupialia).

Species predictive posterior distributions from cross validation for all samples (except two ovicaprids) are visualized in Fig. 7A which shows posterior distributions from models trained on spectra ratios from top hit species. Wider, flatter distributions reflect greater uncertainty in the models for these values. All incorrectly identified mammalian samples (numbers 1–9) were poorly represented in the mammalian database (Supplemental Table S5). Numbers 10–16 which cluster to the left side of the figure with the lowest probability of correct species assignment are, as expected, the seven non-mammals.

• *Order and human origin predictions *For top hit order predictions (mammalian database search) and human origin predictions (vertebrate database search) the ROC AUCs of mean predictive posteriors from cross validation were 1, indicating very accurate predictions of correct order and human origin.

Mean probability of correct order identification for correct samples was 0.97 ± 0.04 and mean probability of correct order identifications for incorrectly identified samples was 0.15 ± 0.24 (Fig. 7B). Order predictive posterior distributions from cross validation for all samples are visualized in Fig. 7B. Correctly identified samples cluster closely to the right while non-mammals cluster to the left. The only exception, Virginia opossum, stretches from the bottom right (orange) to the center of the figure.

Mean probability of human origin for human samples (vertebrate database search) was 0.94 ± 0.03 and mean probability of human for non-human samples was 0.03 ± 0.12 (Fig. 7C). The one non-human primate tested (rhesus monkey, *Macaca mulatta*) had a human spectra ratio of 0.68 with a 0.77 probability of being human, showing clear separation of *Macaca* from human samples, for which the lowest mean probability returned in cross validation was 0.86. Human origin predictive posterior distributions from cross validation for all samples are visualized in Fig. 7C. Human samples cluster closely to the right and are clearly separated from non-human samples that cluster the left. The only exception, rhesus monkey, stretches from the bottom right (orange) to the center of the figure.

##### Models trained on spectra ratios from all available samples

**Figure 8:**
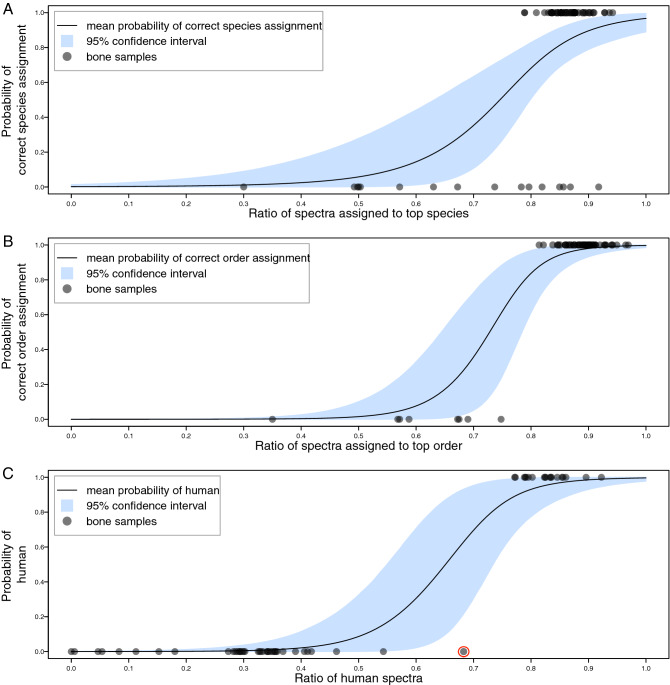
Models of correct species, order and human identifications. Logistic regression models shown in (**A**–**C**) are generated using all available sample data in order to illustrate posterior probability distributions for a range of possible spectra ratios. (**A**) Logistic regression model for probability of correct species identification using spectra ratio, trained on mammalian bone samples of known species and all non-mammal bone samples using a mammalian database. Ratios of correctly identified samples are at y = 1, misidentified samples at y = 0. Mean probability of correct species identification at all possible spectra ratios (0–1) is shown within a 95% confidence interval. (**B**) Logistic regression model for probability of correct order identification using spectra ratio, trained on mammalian bone samples of known order and all non-mammal bone samples using a non-mammalian database. Ratios of correctly identified samples are at y = 1, misidentified samples at y = 0. Mean probability of correct order identification at all possible spectra ratios (0–1) is shown within a 95% confidence interval. (**C**) Logistic regression model for probability of human identification using spectra ratio, trained on known human and non-human bones samples searched against a vertebrate database and ratios of human assigned spectra to total spectra determined. Ratios of human samples are at y = 1, non-human samples at y = 0. Mean probability of human identification at all possible human spectra ratios (0–1) is shown within a 95% confidence interval. The one non-human primate tested (rhesus monkey, *Macaca mulatta*) had a human spectra ratio of 0.68, showing clear separation of *Macaca* from human samples (circled red).

Logistic regression models were trained with all available sample data in order to estimate the posterior probabilities for a range of possible spectra ratios. Distributions and mean values of logistic regression alpha and beta are shown in Supplemental Figure 1 for all three models. Figure 8A shows the average probability of correct species assignment within a 95% CI (confidence interval) over the range of possible spectra ratios (0—1); see also Supplemental Table S9A. Figure 8B shows the average probability of correct order assignment within a 95% CI over the range of possible spectra ratios (0–1); see also Supplemental Table S9C. A comparison of the probability of correct order identifications with correct species identifications (Fig. 8B and A respectively) shows that nearly all samples were correctly identified by order. As expected, the seven non-mammalian samples were incorrectly identified in this mammalian database search. The only mammal not to be correctly identified by order was Virginia opossum (order Didelphimorphia), which was identified as Tasmanian devil (order Dasyuromorphia). Figure 8C shows the average probability of a sample being of human origin within a 95% CI over the range of possible human spectra ratios (0–1); see also Supplemental Table S9A.

## Discussion

The ability to identify an organism from proteins is similar to identifying an organism from DNA. It depends on the amount of protein or DNA available for investigation, the specific sequence(s) being analyzed, the accuracy of sequencing, and the thoroughness of databases. Identifying vertebrates from bone samples using proteins pushes against the limitations of all these criteria. First, the number of proteins routinely extracted from bone with good sequence coverage is typically two, COL1A1 and COL1A2. This is significantly less than the number of proteins that are regularly extracted from other tissues or body fluids which can number in the thousands^15–18^. This paucity of sequencing targets inherently limits the data available for analysis. Making species identification from bone more difficult, is the nature of these two proteins—both are highly conserved over evolution^5^, thus further limiting their informative value with respect to speciation. These problems, the number of proteins available for analysis and their evolutionary conservation, make the third criterion, MS accuracy, even more difficult to overcome—and is the focus of this work. As demonstrated, approximately 40% of all identified peptides from a sample do not identify the bone species from which they came. Some samples have no peptides specific to their species, yet have multiple peptides specific to other species, and some samples have three or more homologous peptides of equal confidence—a highly unlikely event in diploid vertebrates. These data demonstrate that even with spectra quality filters (E-values) for peptide selection, peptide sequence errors (see above) are sufficient to confound species identification from bone. While some of the “misidentified” peptides may be true, unrecognized, polymorphisms, the bulk are unlikely to be so. As already discussed, peptides that identify a species other than the sample species are often species homologous peptides with one or more isobaric amino acid changes, or are the result of post-translational modifications, or the consequence of amino acid sequence inversion where no fragment ion is detected that can distinguish them. These “errors”, although reasonable alternative sequence interpretations of peptides with similar E-values, are numerous, misidentify samples, cannot be eliminated in an unknown sample, and must be considered in species identification. More confounding is the large number of peptides that identify a species other than the sample species and that differ from the sample species peptide by one or more non-isobaric amino acids. Non-isobaric amino acid changes that result from two or more nucleotide changes or multiple amino acid changes in a single peptide are biologically less likely to occur than changes that result from a single nucleotide change^11^, yet many were identified (Fig. 2). And while the mean difference between E-value of single nucleotide and multiple nucleotide or multiple amino acid changes in a single peptide were statistically significant, the use of E-values to eliminate them did not prove beneficial for species identification.

Numerous methods for species identification using bone proteins have been proposed^2,3,6,10^. Many rely on detection of species-specific peptide(s) to make these determinations. However, as demonstrated here, the frequency of identifying high quality, spurious, homologous peptides from species other than the sample species inherently limits the usefulness of these methods as exemplified by the identification of the goat-specific peptide GPSGEPGTAGPPGTPGPQGFLGPPGFLGLPGSR^10^ in deer sample 28 (Supplemental Tables S5 and S9). Additionally, as more species are sequenced, and as more single amino acid polymorphisms are detected, peptide uniqueness diminishes, a phenomenon known as signal erosion^19^ which inevitably reduces the accuracy of species identification.

Progress in microbial species and toxin identifications have broadened the concept of species-specific peptides to a “strong peptide” hypothesis in which specific peptides are believed to be “immune to signal erosion”^1^—i.e. that there is a diminished likelihood that a specific group of “strong peptides” from one organism will evolve and identify another organism. And, if properly weighted, strong peptides can add statistical power to species determination. When large numbers of proteins are available, this method may be sufficient, however, when few proteins are available for analysis, and they are highly conserved, as in bone, this method may suffer from the same limitations as the unique peptide method—particularly in light of high quality spectra that identify erroneous peptides^6,8^. In this regard the present method, which is unbiased, considers all data, and does not weigh specific peptides, should also prove useful for species identification when proteome datasets are larger—e.g. from animal and plant tissues, and perhaps, microbial homogenates.

## Methods

### Materials

Chemical reagents including salts, buffers, detergents, organics, alpha-cyano-4-hydroxycinnamic acid and Bradford protein assay kits were purchased from Sigma (St. Louis, MO). Trypsin Gold was from Promega (Madison, WI). C18 ZipTips (Millipore, MA) were purchased from Thermo Fisher Scientific, Waltham, MA. All bone samples were from the osteological teaching collection of the Department of Anthropology in New York City Office of Chief Medical Examiner. Laboratory work was performed at the New York City Office of Chief Medical Examiner. In some cases, a single bone was sampled in multiple locations. Detailed information about samples (including bone number and sample number) may be found in Supplemental Table S2.

### Extraction

Bone samples (80–200 mg) were fragmented into small pieces and demineralized overnight in ten volumes of 1.2 M hydrochloric acid at 4 °C. Following centrifugation at 13,000*g* for 5 min, one of two methods (Supplemental Table S2) were used to extract protein from the acid insoluble bone precipitate: (i) incubation in 50 mM ammonium bicarbonate (ABC) for 3 h at 65 °C, and (ii) incubation in 50 mM ABC, 8 M Urea for 72 h at 4 °C. Samples were spun at 18,000*g* for 30 min at 4 °C. Supernatant were collected and protein concentrations measured by Bradford using bovine serum albumin as standard.

### In-solution protein digestion

Approximately 20 μg of solubilized bone proteins were reduced in 5 mM Tris(2-carboxyethyl)phosphine hydrochloride (brought to ~ pH 8 with NaOH) for 15 min. Proteins were then alkylated with 15 mM iodoacetamide (IAA) for 15 min and excess IAA quenched with 15 mM dithiothreitol. Fifty mM ABC was added to reduce the urea/ABC solubilized samples to 2 M urea. A 1:20 (wt/wt) ratio of trypsin to sample was digested overnight at 37 °C. Following digestion, samples were dried under vacuum and resuspended in 2% ACN, 0.1% TFA in HPLC water.

### HPLC

Approximately 2 μg of digested protein were separated by nano-HPLC reverse phase chromatography (Dionex Ultimate 3000 LC System, Sunnyvale, CA). Tryptic peptides were desalted using an inline Acclaim Pep-Map100 μ-pre-column trap (C18, 5 μm, 100 Å beads in a 300 μm i.d. × 5 mm column, Dionex) and then separated with a 40 min linear 5–40% ACN gradient on an Acclaim PepMap100 column (C18, 3 μm, 100 Å, 75 μm i.d. × 15 cm, Dionex) at a flow rate of 300 nl/min. ACN gradients were prepared by mixing solvent A (2% acetonitrile, 0.1% TFA in HPLC water) with solvent B (98% acetonitrile, 0.1% TFA and 2% HPLC water). Sample spotting on MALDI plates included inline (1:1) mixing of sample with HCCA matrix (5 mg HCCA/ml 75% ACN and 0.1% TFA in HPLC water). For the 40 min program 96 spots were collected.

### Mass spectrometry data acquisition

MS data were acquired on a 4800 MALDI TOF/TOF (AB Sciex, Framingham, MA) at a laser repetition rate of 200 Hz with 600 laser shots/spectrum (50 laser shots/sub-spectrum). MS/MS data were acquired at 200 Hz in 2 kV MS/MS mode with 2250 laser shots/spectrum (50 laser shots/sub-spectrum) with the following TOF/TOF Series Explorer Stop Conditions: maximum shots per spectrum 2,250, minimum shots per spectrum 800, number of MS/MS fragments 8, with a signal/noise ratio of each fragment 75. Typically, the top 30 strongest peaks were selected for MS/MS analysis. Raw data were transformed to mascot generic format using ProteinPilot 4.5 (Sciex).

### Mass spectrometry data processing

LC–MS/MS data were processed using X! Tandem (http://p3.thegpm.org/tandem/thegpm_ppp.html) and searched against NR mammalian and vertebrate databases (2016 March) (http://www.ncbi.nlm.nih.gov) with at most 1 missed cleavage, precursor mass error ± 250 ppm and fragment mass error 0.3 Da. Each peptide spectrum match (PSM) was assigned a score and a corresponding E-value, which indicates the number of peptides that would be expected to achieve at least that score for a given mass spectra and database^9^. X! Tandem output xml files included all peptides identified with E-value < 0.1 and possible proteins of origin. PSMs were filtered for an overall false discovery rate (FDR) of < 1%, which was estimated using a decoy database of reversed target protein sequences searched simultaneously with target database and calculated per sample as the total non-redundant (or unique) decoy PSMs divided by the total non-redundant target PSMs. Samples were filtered for the E-value that achieved < 1% FDR or E-value 0.01 if lower. The additional E-value filter was applied because many samples had no decoy hits, or only one or two with high E-values (> 0.01).

### Pairwise comparison of bone sample spectra

A similarity ratio was calculated for all pairs of bones samples using shared and not shared spectra. For any given pair of samples X and Y, a spectrum x^i^ was considered shared if any of the sequences matching x^i^ are also found in Y. The similarity ratio of X and Y is then calculated as (x_shared_ + y_shared_)/(X_T_ + Y_T_) where x_shared_ and y_shared_ are the number of shared spectra in X and Y, respectively, and X_T_ and Y_T_ are the total number of spectra in each X and Y. This method takes into account the differences in number of spectra identified across the sample set which varies from 79 to 710.

### Determining the probability that a sample is correctly assigned to species, order, or is of human origin

Bayesian probabilistic logistic regression models were fitted to processed search result data from bone samples using Markov chain Monte Carlo (MCMC) simulation (python 3.7, package PYMC3 3.8) (script in Supplemental Data). A Bayesian probabilistic model was chosen for its relative stability with smaller and imbalanced datasets, as well as its ability to generate informative probability distributions rather than point estimates^13^. The model was trained on three different types of search results generated from the same raw MS data from bone samples using:

1. Mammal database for species determination—bone data were searched against the mammal database and tallied for the highest ratio of hits (peptide spectrum match, peptide sequence, or spectra) for a single species to the total number of database hits per sample. The model was trained to determine if this ratio was predictive of whether the resulting “top hit” species matched the known species of origin of the sample.
2. Mammal database for order determination—bone data were searched against the mammal database and tallied for the highest ratio of hits (peptide spectrum match, peptide sequence, or spectra) for a single taxonomic order to the total number of hits per sample, to determine if this ratio was predictive of whether the “top hit” order matched the known taxonomic order of the sample.
3. Vertebrate database for human determination—bone data were searched against the vertebrate database and tallied for the number of hits (peptide spectrum match, peptide sequence, or spectra) to *Homo sapiens* database entries verses the total number of database hits per samples, to determine if this ratio of human hits to total was predictive of *Homo sapiens* as the species of the sample.

For analysis of the accuracy of both species and order matches, the mammal database was chosen to deliberately generate incorrectly identified samples (i.e. all non-mammal samples) in order to train the model. Note, that for analysis of correct species matches the two ovicaprid samples were removed from the dataset as their species of origin is unknown. The five non-mammal samples of unknown species (2 birds, 2 fish, and 1 frog) were still used since any species matches to a mammal database search are incorrect.

For the three sets of search results described above, models were trained separately with peptides, PSMs, and spectra, and compared using a Pareto-smoothed importance sampling approximate of leave-one-out cross-validation (PSIS-LOO, implemented with PYMC3)^14^. This was done to determine if the method of analyzing the search results affected the out-of-sample prediction accuracy of the fitted models. Based on the results of this comparison, subsequent analyses were performed using spectra ratios.

In order to test the performance of each model (species, order, and human) on unseen data, exact leave-one-out cross validation was performed. Each model was iteratively trained on all data except one sample, then the trained model was used to generate the posterior predictive distribution of the unseen sample data.

Finally, models were trained with all available bone sample data (56 bone samples for species, 58 bone samples for order and determination of human origin) in order to generate and visualize posterior probability distributions for the full range of possible spectra ratios (0–1) and estimates of logistic regression parameters alpha and beta for each model.

## Supplementary Information

Supplementary Table S1.

Supplementary Table S2.

Supplementary Table S3.

Supplementary Table S4.

Supplementary Table S5.

Supplementary Table S6.

Supplementary Table S7.

Supplementary Table S8.

Supplementary Table S9.

Supplementary Table S10.

Supplementary Table S11.

Supplementary Table S12.

Supplementary Information.

Supplementary Figure 1.

## Data Availability

The mass spectrometry proteomics data have been deposited to the ProteomeXchange Consortium via the PRIDE^20^ partner repository with the dataset identifier PXD021963. The datasets generated during and/or analyzed during the current study are available as Supplemental Data. Python script for Bayesian logistic regression model is available in Supplemental Data.
