# Supplementary material for: A predictive model for vertebrate bone identification from collagen using proteomic mass spectrometry
Source: Sci Rep. 2021 May 25;11:10900. doi: 10.1038/s41598-021-90231-5 (PMC8149876; doi:10.1038/s41598-021-90231-5)
Supplement: Supplementary file 10 — Supplementary Table S10. [file 41598_2021_90231_MOESM10_ESM.docx]

| **Supplemental Table 10A: Hypothetical peptide sequences - total peptides assigned vs total spectra assigned to species** | | | | |  |
| --- | --- | --- | --- | --- | --- |
| Spectra | Sequence  (Hypothetical) | Species | Number of Peptides | Number of Spectra | |
| A | A1 - VQC**L**D**L**STR | Cat, Rat | 1 | 1 | |
|  | A2 - VQC**I**D**L**STR | Dog | 1 |  |  |
|  | A3 - VQC**L**D**I**STR | Human | 1 |  |  |
|  | A4 - VQC**I**D**I**STR | Pig | 1 |  |  |
| B | B1 - HTVA**Q**CWR | Goat, Rat | 1 | 1 | |
|  | B2 - HTVA**K**CWR | Human, Dog | 1 |  |  |
| C | C1 - TAP**L**V**L**FGK | Cow | 1 | 1 | |
|  | C2 - TAP**L**V**I**FGK | Human | 1 |  |  |
|  | C3 - TAP**I**V**L**FGK | Mouse | 1 |  |  |
|  | C4 - TAP**I**V**I**FGK | Pig | 1 |  |  |
| Total | | | 10 | 3 | |

| **Supplemental Table 10B: Percent hypothetical peptides & spectra assigned per species (from peptides above)** | | |
| --- | --- | --- |
| Species | Total Peptides Assigned (%) | Total Spectra Assigned (%) |
| Cat | 1 (10%) A1* | 1 (33%) A |
| Cow | 1 (10%) C1 | 1 (33%) C |
| Dog | 2 (20%) A2, B2* | 2 (67%) A, B |
| Goat | 1 (10%) B1* | 1 (33%) B |
| Human | 3 (30%) A3, B2*, C2 | 3 (100%) A, B, C |
| Mouse | 1 (10%) C3 | 1 (33%) C |
| Pig | 2 (20%) A4, C4 | 2 (67%) A, C |
| Rat | 2 (20%) A1*, B1* | 2 (67%) A, B |
| *Blue = peptides shared between species | | |

A predictive model for vertebrate bone identification from collagen using proteomic mass spectrometry

Heyi Yang; Erin Butler; Samantha A. Monier; Jennifer Teubl; David Fenyo; Beatrix Ueberheide; Donald Siegel
