# Supplementary material for: A predictive model for vertebrate bone identification from collagen using proteomic mass spectrometry
Source: Sci Rep. 2021 May 25;11:10900. doi: 10.1038/s41598-021-90231-5 (PMC8149876; doi:10.1038/s41598-021-90231-5)
Supplement: Supplementary file 13 — Supplementary Information. [file 41598_2021_90231_MOESM13_ESM.zip › OCMEspecies_logreg/cover page.docx]

**A predictive model for vertebrate bone identification from collagen using proteomic mass spectrometry**

Heyi Yang^†1^; Erin Butler^†1^; Samantha A. Monier^1^; Jennifer Teubl^2^; David Fenyo^2^; Beatrix Ueberheide^2^; Donald Siegel^1*^

1Office of Chief Medical Examiner, New York, NY; ^2^NYU Medical Center, New York, NY

†Authors contributed equally to this work. *Corresponding author [DSiegel@ocme.nyc.gov](mailto:DSiegel@ocme.nyc.gov)
