# Supplementary material for: A predictive model for vertebrate bone identification from collagen using proteomic mass spectrometry
Source: Sci Rep. 2021 May 25;11:10900. doi: 10.1038/s41598-021-90231-5 (PMC8149876; doi:10.1038/s41598-021-90231-5)
Supplement: Supplementary file 14 — Supplementary Figure 1. [file 41598_2021_90231_MOESM14_ESM.pdf]

A predictive model for vertebrate bone identification from collagen using proteomic mass spectrometry

Heyi Yang[†1]; Erin Butler[†1]; Samantha A. Monier[1]; Jennifer Teubl[2]; David Fenyo[2]; Beatrix Ueberheide[2]; Donald Siegel[1*]

[1]Office of Chief Medical Examiner, New York, NY; [2]NYU Medical Center, New York, NY

†Authors contributed equally to this work. *Corresponding author DSiegel@ocme.nyc.gov

Distributions of alpha and beta logistic regression parameters for trained logistic regression models. Histograms show the distributions and means of logistic regression model parameters alpha and beta for trained models predicting A) probability of correct species assignment using spectra ratio, B) probability of correct order assignment using spectra ratio, and C) probability that sample is of human origin using human spectra ratio. (See Methods for details.)

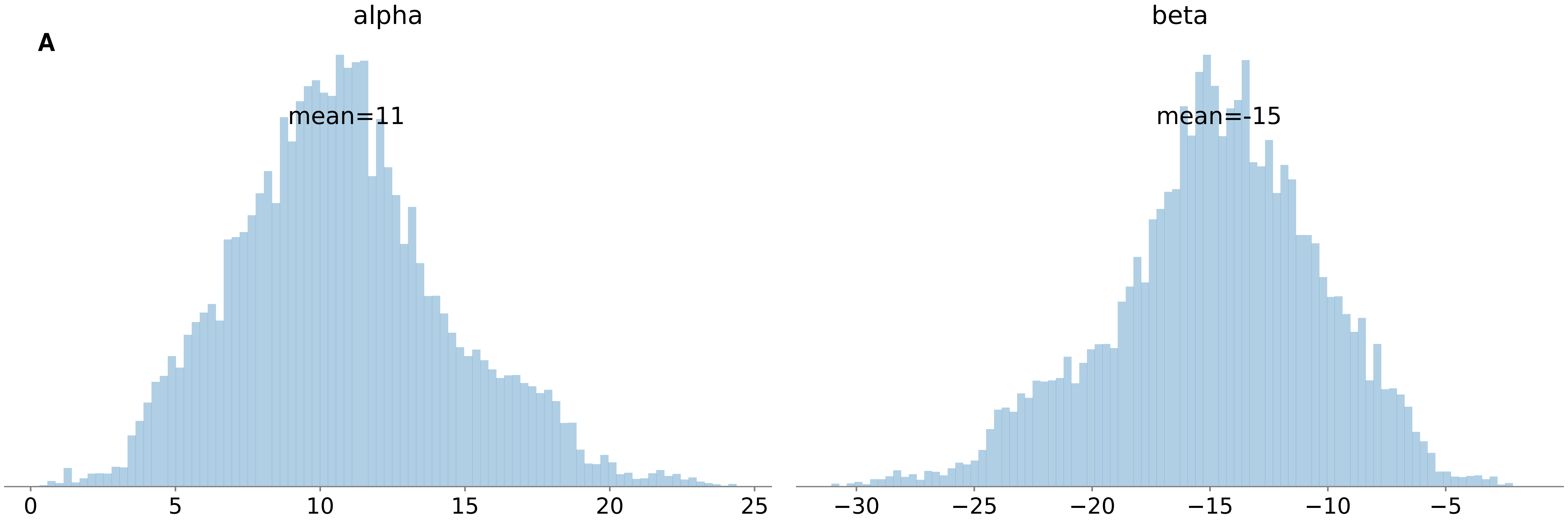
A
alpha
mean=11
0
5
10
15
20
25
beta
mean=-15
−30
−25
−20
−15
−10
−5

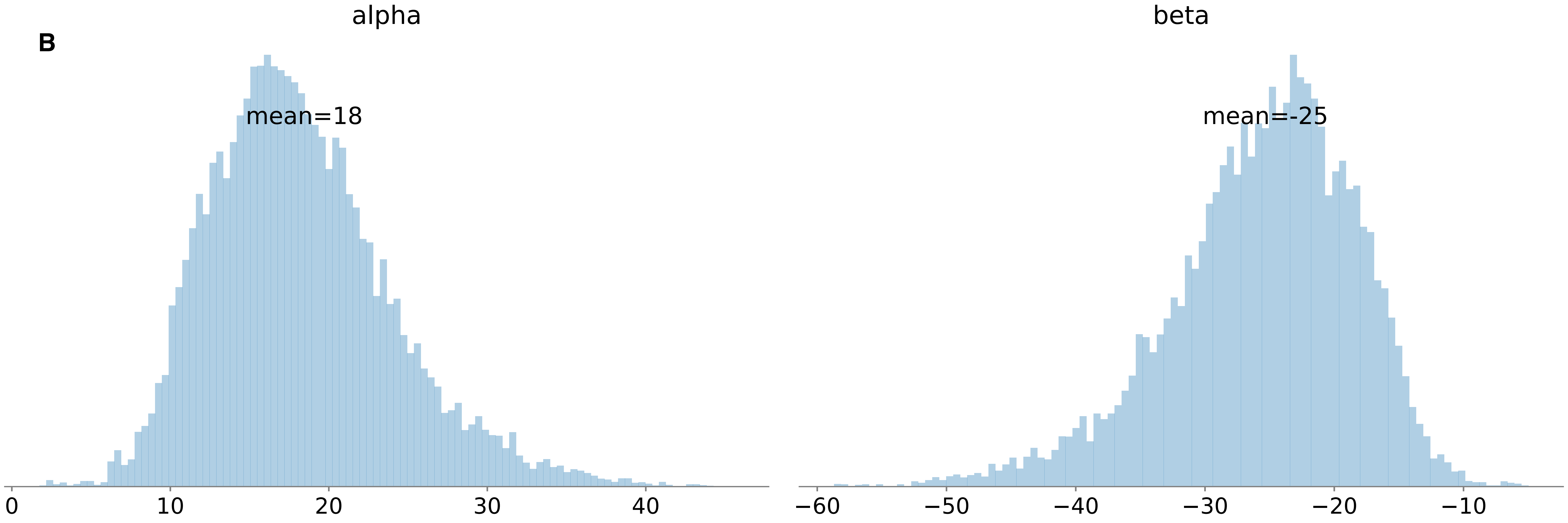
B
alpha
mean=18
0
10
20
30
40
beta
mean=-25
−60
−50
−40
−30
−20
−10

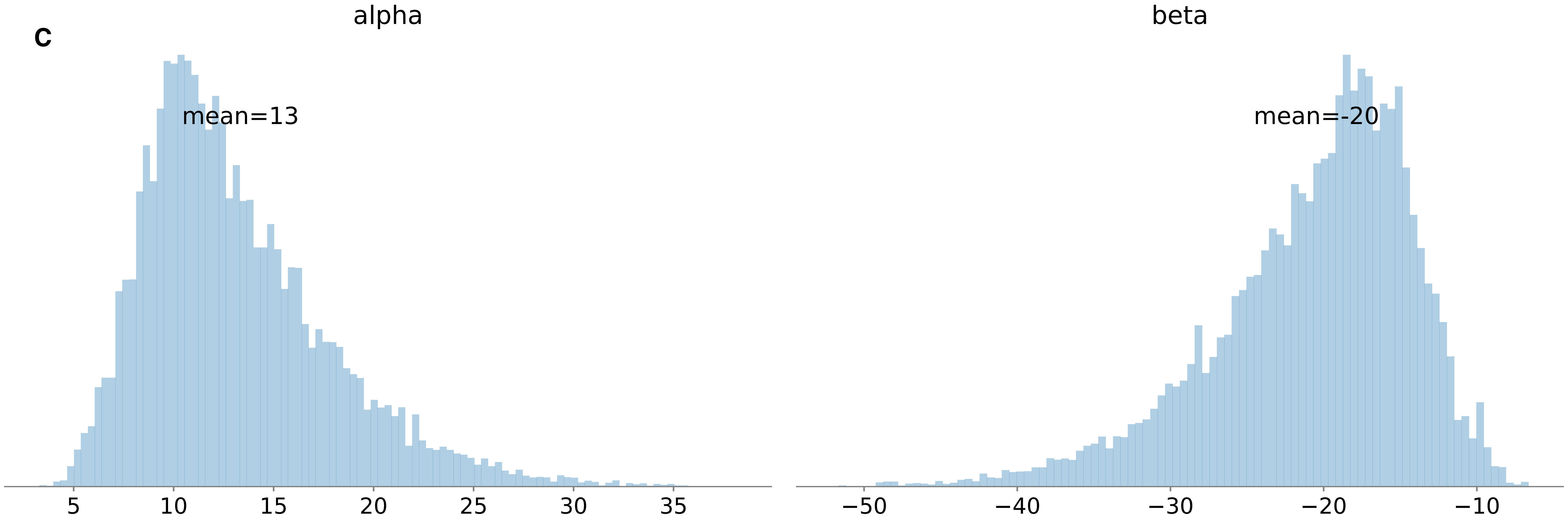

c
alpha
mean=13
5
10
15
20
25
30
35
beta
mean=-20
−50
−40
−30
−20
−10
